# The Social Contexts of Birthing People with Public- and Private-Payer Prenatal Care: Illuminating an Understudied Aspect of the Patient Experience

**DOI:** 10.1089/heq.2021.0168

**Published:** 2022-12-12

**Authors:** Sarah B. Garrett, Melissa A. Simon

**Affiliations:** ^1^ Philip R. Lee Institute for Health Policy Studies, University of California, San Francisco, California, USA.; ^2^ Feinberg School of Medicine, Northwestern University, Chicago, Illinois, USA.

**Keywords:** patient perspective, social context, patient knowledge, cultural humility, prenatal care, person-centered research

## Abstract

**Purpose:**

In pursuit of more equitable and person-centered health care, patients and professional medical societies increasingly call for better clinician understanding of patients' perspectives and social contexts. A foundational but understudied aspect of patients' social contexts are the ideas they encounter about health-related behaviors. We investigated this aspect of the social contexts of birthing people, comparing those with public versus private insurance to discover setting-specific insights.

**Methods:**

Based on ethnographic fieldwork, we created an original survey featuring 29 statements about 12 prenatal, perinatal, and postpartum health behaviors (e.g., drinking alcohol, epidural use, breastfeeding). Participants were 248 individuals receiving prenatal care in Northern California in 2009–2011, split evenly between public- and private-payer coverage. Participants reported whether they were familiar or unfamiliar with each statement.

**Results:**

Ninety-eight percent of all participants had heard contradictory ideas about ≥1 health behavior (mean=3.9 behaviors for public- and 5.4 for private-coverage respondents). For 20 of the 29 behavior-related ideas, exposure varied significantly by coverage type. Among other differences, public-coverage respondents were much more familiar with ideas related to risk and constrained autonomy (e.g., that serious perinatal complications are common; that new mothers should try to breastfeed even if they do not want to).

**Conclusions:**

Birthing people are exposed to a wide range of ideas about health behaviors, many of which vary by the structural systems in which they are embedded. Understanding and engaging this complexity can help clinicians to provide more respectful, person-centered, and equitable maternity care.

## Introduction

Professional organizations and reproductive health scholars increasingly advocate for a better understanding of the social contexts of birthing people.^1–7^ With the goal of improving maternal health care and outcomes, entities including the National Academies have called for research on new topics and outcomes that “move beyond traditional clinical variables,”^3^ particularly for communities disproportionately burdened by adverse birth outcomes.^2,4^ Scholars have advocated for more nuanced research on how pregnant individuals perceive and evaluate the world around them.^1,8–11^

Advancing research in this area may be particularly important for individuals disproportionately burdened by maternal health inequities. Non-Hispanic Black women, American Indian/Alaska Native women and, regionally, select other racial/ethnic groups experience disproportionate rates of maternal and pregnancy-related mortality and morbidity.^12–15^ Many experience problems of patient–provider communication and interaction^7,16–20^ and want clinicians to better understand them and their experiences.^5,21–23^ Limited data exist, however, that illuminate the different social contexts of birthing people. Survey data that document decision making, outcomes, or care quality reflect narrow aspects of their worlds,^19,24–26^ while holistic social science ethnographies of pregnant and birthing people^27–30^ typically focus on single settings or social groups.

One way to better understand patients' social contexts is to understand the health- and health care-related ideas they encounter—ideas that they bring with them into their appointments. Referencing examples from qualitative research on breastfeeding, a new mother might believe that breastmilk is best for her baby, but she may also have heard that formula is just as good, or that formula may be healthier than breastmilk.^31–33^ The ideas that individuals encounter are foundational to their decision making and broader interpretation of the world, informing their view of what is possible, likely, or under debate.^34–37^

Health services and public health scholarship sometimes addresses individuals' preferences and beliefs.^38–40^ However, there is very little research on individuals' exposure to health-related ideas despite experimental data suggesting considerable influence.^41–44^ (Research on health “misinformation” is a current exception.)^44–46^ Our review finds no scholarship that systematically examines the health- and health-care related ideas birthing people encounter. We sought to address this gap.

The purpose of this study was to generate novel information about ideas pregnant individuals encountered about prenatal, perinatal, and postpartum behaviors. Following recent work,^24^ we explored similarities and differences between patients with public versus private insurance coverage. We paid special attention ideas related to patient autonomy and risk, as these are widely documented in the experiences of individuals disproportionately burdened by poor maternal health outcomes.^7,16,18–20,27,47^ Understanding how patients think about the pre-, peri-, and post-natal period can help clinicians to provide more respectful, person-centered, and equitable maternity care.

## Data and Methods

The data presented here were collected as part of a large observational, longitudinal, multistage mixed methods^48^ study on birthing people's preferences, perspectives, and experiences. Respondents completed up to three online- or paper-based self-report surveys in English or Spanish as they transitioned from pregnancy through 2–3 months postpartum. The data in this article were collected in the pre-birth surveys using both existing and novel instruments.

### Setting and participants

From late 2009 through Spring 2011 the lead author and a research team collected longitudinal survey data from pregnant individuals recruited from five Northern California hospital-based clinics (two public, three private), two free-standing community clinics (one public, one private), a large online parenting email group, and a home-birth email group. We purposively selected these settings to access individuals who were receiving care at diverse institutions and who in the aggregate had a wide range of social, educational, and material resources.^49^ Individuals were eligible if they were English or Spanish speaking, age 18 or older, and identified themselves as pregnant. The University of California (UC) Berkeley and UC San Francisco Institutional Review Boards approved the study.

### Measures

#### Exposure to ideas about perinatal care and behaviors

Our literature review identified no published survey instruments on pregnant individuals' exposure to diverse perspectives or ideas about pregnancy, birth, and new parenthood, so the lead author conducted formative qualitative research to develop an original instrument.^38,50–53^ She spent 6 months observing prenatal and new parent classes and conducted over a dozen expert interviews with obstetricians, midwives, labor and delivery nurses, and doulas. From this research she (1) identified ideas patients and providers referenced about pre-, peri-, and post-partum topics; (2) selected focal topics about which there were multiple different ideas that might guide individuals' decisions and behaviors; and (3) drafted short statements representing the observed patients' or practitioners' ideas about those topics.^50^

For example, the instrument refers to “All in all, epidurals are *good* for birthing women,” and “All in all, epidurals are *bad* for birthing women.” Items were ordered randomly in the survey and, for each, the respondent was asked whether they had heard of the idea (*Yes, No, I don't know*). Drawn from the experiences of patients and practitioners, some queried ideas reflect medical advice while many do not. Reflecting the language these individuals used, the survey refers to “women” rather than the non-gendered language used in this article. Following survey development best practices, the lead author and research assistants conducted multiple rounds of pre-testing with linguistically-, racially-, ethnically-, and socioeconomically diverse women, and revised the instrument for flow and clarity.^54,55^

The final survey represents 29 distinct ideas about 12 topics (Table 1). Pre-testers reported that the instrument represented a variety of familiar and unfamiliar perspectives, indicating both face and content validity for an instrument designed to represent diverse ideas. More information about the survey's design and validation in the study sample is available elsewhere.^56^

**Table 1.** List of Surveyed Ideas by Topic

A. Physical activity during pregnancy
That women should become *less* physically active while they are pregnant.
That women should *maintain* their normal level of physical activity while they are pregnant.
That women should become *more* physically active while they are pregnant.
B. Factors that affect fetal health
That an unborn baby's health depends on *God more than anything else*.
That an unborn baby's health depends on *genetics more than anything else*.
That an unborn baby's health depends on the *mother's actions more than anything else*.
C. Experience of pregnancy
That pregnancy itself is a great experience.
That there is nothing great about pregnancy except for the baby.
D. Drinking during pregnancy
That it is *OK* for a pregnant woman to drink a glass of wine or beer every now and then.
That it is *good* for a pregnant woman to drink a glass of wine or beer every now and then.
That it is *never OK* for a pregnant woman to drink a glass of wine or beer.
E. Epidural use
That, all in all, epidurals are *good* for women in labor.
That, all in all, epidurals are *bad* for women in labor.
F. Risk in labor and delivery
That it is *common* for women to have serious complications during labor and delivery.
That it is *rare* for women to have serious complications during labor and delivery.
G. The value of breastmilk vs. formula
That breastmilk and formula are *equally good* for infants.
That *breastmilk* is better than formula for infants.
That *formula* is better than breastmilk for infants.
H. The acceptability of nursing boy vs. girl babies
That it is more acceptable to nurse a *boy baby* than a girl baby.
That it is more acceptable to nurse a *girl baby* than a boy baby.
That it is *equally acceptable* to nurse girl and boy babies.
I. The timing of baby feeding
That babies should be fed on a *set schedule*.
That babies should be fed *whenever they seem hungry*.
J. Breastfeeding as compulsory or optional
That a new mother should try to breastfeed *even if she does not want to*.
That if a new mother does not want to breastfeed, *that is a good enough reason for her not to*.
K. Experience of birth
That giving birth is an *empowering* experience.
That giving birth is an *embarrassing* experience.
L. Babies' ability to manipulate
That young babies try to manipulate their parents *on purpose*.
That young babies *cannot* try to manipulate their parents on purpose.

#### Insurance type

The demographics survey asked respondents about the type of coverage they had for prenatal and birth care: Medi-Cal, private, or None. Medi-Cal is California's Medicaid program, for which pregnant individuals with incomes at or below 200% of the federal poverty level were eligible. Respondents with missing insurance information who received care at public safety-net clinics were coded as receiving Medi-Cal.

#### Other demographics

Race and ethnicity was queried in one question about which single category “best describes your race or ethnic group.” Age was reported at time of survey then calculated to reflect age at a common timepoint across the sample (December 31, 2009). Highest level of educational achievement was assessed in one of four categories.

### Analysis

Descriptive statistics were used to evaluate differences between the payer groups: Chi square and Fischer's exact test for categorical data, and *t*-tests to evaluate differences in mean age, a normally distributed interval variable.

## Results

### Sample description

Of the 325 pregnant individuals who began the survey, 248 completed over 90% of the questions and had data on coverage type. Approximately half of respondents with public coverage had missing data on education, 16% had completed high school, 24% some college, and nearly 12% a college or graduate degree (Table 2). Twenty-seven percent identified themselves African American or Black, 27% as Latina/Hispanic, 16% as white, 11% as Asian/Pacific Islander, and 8% as multiple or other race/ethnicity; 10% did not report. Fifty-two percent were first-time mothers. Seventeen percent chose the Spanish-language survey. One-third of the sample had data on age (mean=28 years, standard deviation [SD] 6.2).

**Table 2.** Sample Description by Coverage Type

	Insurer	
	Public (***n***=124)	Private (***n***=124)
Level of education^*^		
High school or less	20 (16.1%)	0 (0.0%)
Some college	30 (24.2%)	7 (5.7%)
College degree	11 (8.9%)	41 (33.1%)
Graduate degree	3 (2.4%)	76 (61.3%)
Missing	60 (48.4%)	0 (0.0%)
Race/ethnicity^a,^^*^		
African American/Black	34 (27.4%)	1 (0.8%)
Asian/Pacific Islander	14 (11.3%)	13 (10.5%)
Latina/Hispanic	34 (27.4%)	2 (1.6%)
Multiple or other	10 (8.1%)	5 (4.0%)
White	20 (16.1%)	102 (82.3%)
Missing	12 (9.7%)	1 (0.8%)
Spanish-language survey^*^	21 (16.9%)	0 (0.0%)
First-time mother		
Yes	65 (52.4%)	71 (57.3%)
Missing	3 (2.4%)	0 (0.0%)
Mean age in years^b,^^*^ (SD)	27.9 (6.2)	33.6 (4.5)
Missing	79 (63.7%)	0 (0.0%)

Note: Categories with an asterisk (^*^) indicate variables differ significantly by payer (*p*<0.05).

^a^ Respondents were asked to select a single category.

^b^ Age on December 31, 2009.

SD, standard deviation.

Among those with private insurance, nearly 6% had completed some college, 33% a college degree, and 61% a graduate degree. Eighty-two percent identified themselves as white, nearly 11% as Asian/Pacific Islander, 4% multiple or other race/ethnicities, <2% as Latina/Hispanic, and <1% as African American or Black; <1% reported no race/ethnicity data. Fifty-seven percent were first-time mothers. Mean age was nearly 34 years (SD 4.5). All answered the English-language survey. Respondents were not asked about gender, so we refer to them in non-gendered terms.^57^

### Exposure to ideas by payer group

More than 98% of participants were familiar with multiple ideas about one or more surveyed topics, reflecting exposure to contradictory ideas about prenatal, perinatal, and postpartum behaviors. Across the 12 topics, public-payer respondents had heard two or more ideas about 3.9 topics (SD 2.2), private-payer respondents about 5.4 topics (SD 2.4; *p*<0.00; not shown). Exposure to contradictory ideas varied by topic, ranging in the public-payer group from 6.5% (nursing girl vs. boy babies) to 62.9% (physical activity during pregnancy) and in the private-payer group from 0.8% (nursing girl vs. boy babies) to 87.1% (timing of baby feeding; Appendix Table A1).

Of the 29 ideas surveyed, eight were more familiar to those with public coverage and 12 more familiar to those with private. Similar proportions of each group recognized nine of the ideas.

#### Prenatal topics (topics A–D)

**FIG. 1.**
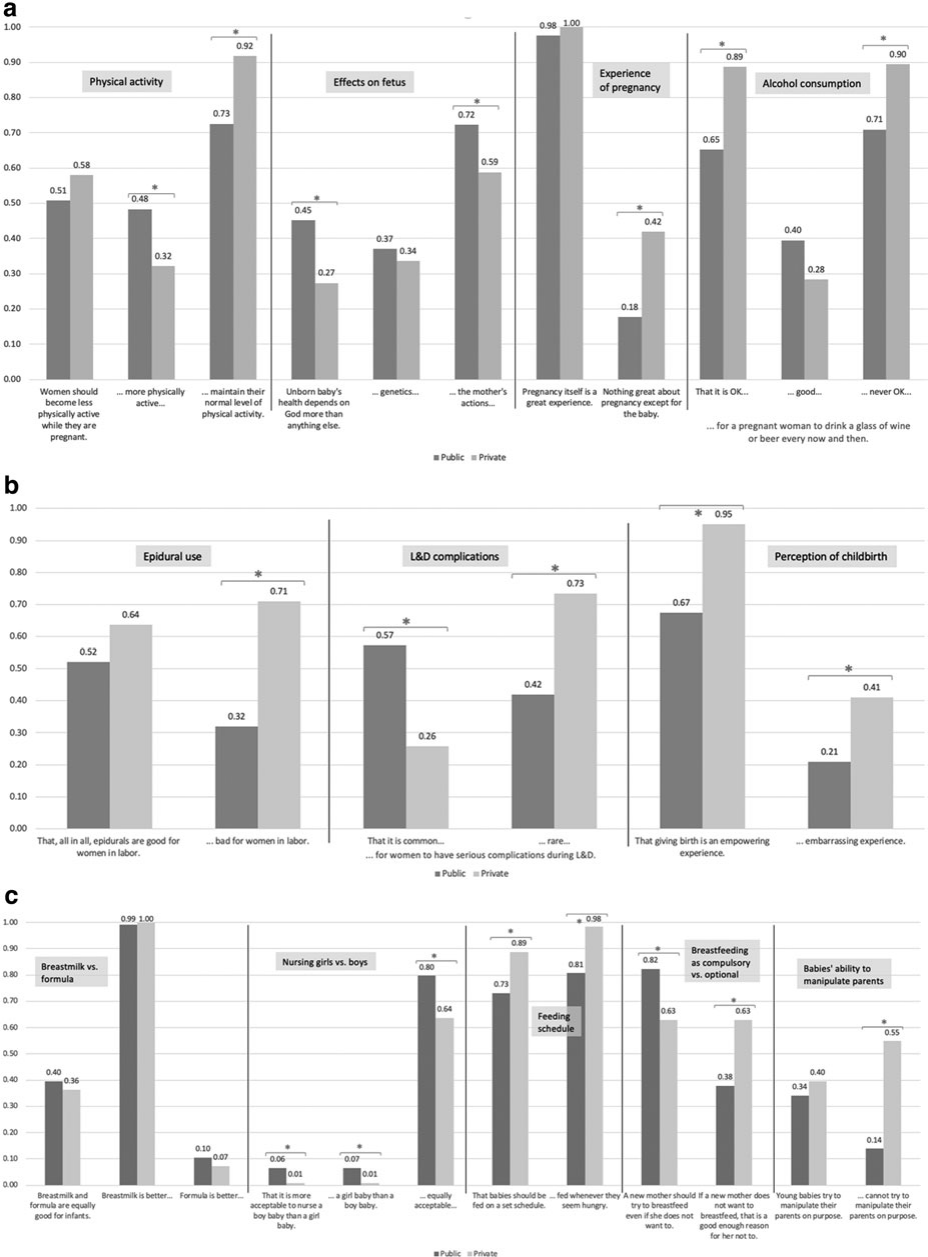
**(a)** Proportion of respondents familiar with surveyed ideas, by insurance coverage type: Prenatal topics. **(b)** Proportion of respondents familiar with surveyed ideas, by insurance coverage type: Perinatal topics. **(c)** Proportion of respondents familiar with surveyed ideas, by insurance coverage type: Postpartum topics. Note: Bars with an asterisk (*) indicate familiarity differs significantly by payer (*p*<0.05). For complete wording of the statements see Table 1.

Regarding physical activity, public- and private-payer respondents reported similar levels of exposure to the idea that “women should become less physically active while they are pregnant” (Fig. 1a). However, those with public coverage were significantly more likely to have heard that women should “become more physically active,” while private coverage respondents were significantly more likely to have heard that “women should maintain their normal level of physical activity.” Regarding factors that affect an unborn baby's health, similar proportions had heard that genetics were the primary influence. However, public-coverage respondents were more familiar with the other two influences (it “depends on God” or “on the mother's actions”).

Nearly 100% of both groups had heard that “pregnancy itself is a great experience.” In contrast, private-coverage respondents had been significantly more exposed to the idea that there is “nothing great about the pregnancy except for the baby.” Finally, regarding alcohol consumption, small proportions of both groups reported having heard that it was “good” for a pregnant woman to drink a glass of wine or beer. Private-coverage respondents were significantly more likely than their counterparts to have heard that it was “OK” or “never OK” to do so.

#### Perinatal topics (topics E, F, K)

Similar proportions of each group had heard that “all in all, epidurals are good for women in labor” (Fig. 1b). However, private patients were significantly more likely to have heard that epidurals were “bad” for women in labor. The two groups differed markedly in their exposure to ideas about risk in labor and delivery (L&D; discussed below). Private-coverage respondents were significantly more likely to have heard both surveyed ideas about the character of childbirth: that giving birth is “empowering” and “embarrassing.”

#### Postpartum topics (topics G–J, L)

Similar proportions of the groups had heard surveyed ideas about the value of breastmilk and formula: that they were “equally good for infants,” that one was better than the other, and vice versa (Fig. 1c). Very few respondents had heard ideas about the acceptability of nursing girl versus boy babies (e.g., that it's equally acceptable or more acceptable to nurse one gender over another), but public-coverage respondents were significantly more likely to have heard them. In contrast, significantly more private- than public-coverage respondents had heard ideas about the pacing of feeding babies (feeding on a set schedule and feeding flexibly).

The two groups showed marked differences in exposure to ideas regarding breastfeeding as compulsory or optional (see below). Finally, similar proportions of the groups had heard that young babies try to manipulate their parents on purpose. Private-coverage respondents, however, were significantly more exposed to the idea that young babies “cannot try to manipulate their parents on purpose.”

### Exposure to ideas about risk and autonomy

**APPENDIX FIG. A1.**
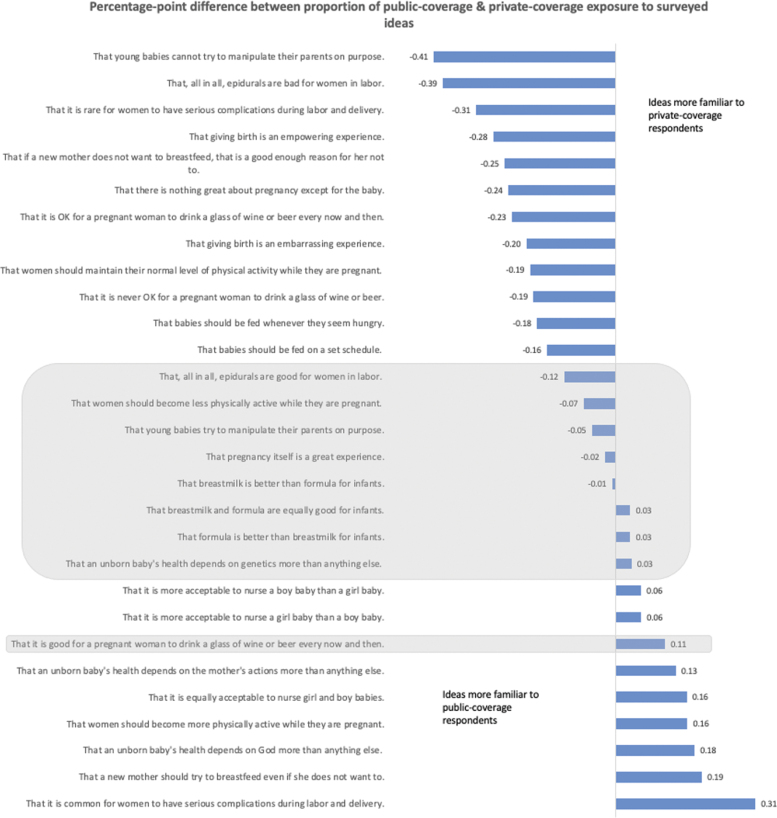
Percentage-point difference between proportion of public- and private-coverage patient exposure to surveyed ideas. Note: All differences are significant in bivariate analyses (*p*<0.05) except those highlighted in gray.

Public- and private-payer groups differed greatly in their exposure to topics related to risk and autonomy (Appendix Fig. A1).

#### Risk: the commonness of serious complications in labor and delivery (topic F)

Public-coverage respondents were approximately twice as likely as their counterparts to have heard that “it is common for women to have serious complications during labor and delivery.” Private-coverage patients were significantly more likely to have heard that “it is rare…” More public-coverage respondents had heard that L&D complications were common than had heard that they were rare. The inverse was true for private-coverage respondents.

#### Autonomy: whether a new mother should try to breastfeed “if she does not want to” (topic J)

Respondents with public coverage were significantly more likely than private-coverage respondents to have heard that a new mother “should try to breastfeed even if she does not want to.” In contrast, respondents with private coverage were significantly more likely to have heard that “if a new mother does not want to breast-feed, that it is a good enough reason for her not to.” Approximately twice as many public-coverage respondents had heard that a new mother should try to breastfeed even if she does not want to than had heard that not wanting to breastfeed was good enough reason not to. In contrast, 6 of 10 private-coverage respondents had heard either idea.

## Discussion

In this article we used novel data to advance understanding of the social contexts of birthing people. We found that the participants—pregnant individuals in Northern California—had encountered many contradictory ideas about consequential health- and health-care-related behaviors by the end of their pregnancies. We found that public- and private-coverage participants had been exposed to some ideas at similar rates (e.g., regarding the relative values of breastmilk vs. formula) but varied significantly in exposure to most of the surveyed ideas.

These differences were particularly pronounced for ideas related to individual autonomy and risk. Far more public- than private-coverage respondents had heard that women “should try to breastfeed even if she does not want to,” and that L&D complications were common. In contrast, far more private-coverage respondents had heard that not wanting to breastfeed “was good enough reason for her not to,” and that serious L&D complications were rare. Based on our review of the literature, this represents the only large-scale survey on pregnant individuals' exposure to ideas encountered on the path toward parenthood.

“Information overload,” specifically regarding exposure to divergent or contradictory ideas about parenting, has been well-documented in the lives of high-socioeconomic status white women.^30,58,59^ Our findings document this across a wider range of topics and populations. Though exposure to contradictory ideas is more extreme among private patients, public-coverage patients experience it as well. Prenatal and postpartum patients across clinic types may benefit from help navigating contradictory information.

Our findings represent both statistically- and clinically significant differences in the ideas public- versus private-coverage patients in Northern California encountered as they moved through pregnancy. The specific ideas and patterns documented in these data, now 10 years old, may have changed. For example, due to popular press coverage, many more individuals today are likely aware of risk in labor and delivery.^60,61^ Importantly, however, the systems and social structures that expose people in different socioeconomic strata to different ideas and experiences persist. Our findings are a reminder that clinicians, whose social backgrounds are historically more similar to those of private-payer patients,^62^ may be unfamiliar with many ideas that patients with public coverage bring with them into their appointments.

Patient experiences of risk and limited autonomy in maternity care are well documented, particularly in Black, indigenous, and other people of color (BIPOC) communities.^13,14,16,17,19^ Due to structural racism, BIPOC individuals are overrepresented among the uninsured^63^ and at institutions providing low-quality care, which perpetuates disparities in maternal mortality and morbidity.^12,20,64^ BIPOC maternity care patients experience disempowerment, coercion, and adverse outcomes,^7,16,18,27,65–67^ and Californians with public coverage experience less control than private patients over important aspects of their hospital birth experience (e.g., choice of provider, consultation before episiotomy; pressure to have a primary cesarean).^24^

Our findings add to these insights, documenting public-coverage respondents' greater exposure to ideas of risk and diminished autonomy in the perinatal period, even among individuals who had not yet experienced birth or early parenthood themselves. These differences likely reflect both different ideas circulating in the social networks of individuals with different socioeconomic statuses,^56^ as well as inequitable experiences that birthing people may have in safety-net versus private clinics and hospitals.^7^

Traditional medical appointments and medical history-gathering methods limit clinicians' ability to learn about their patients. The findings presented here illustrate how greatly patient perspectives can vary across settings and diverge from medical advice, highlighting both the need and challenge of meeting patients where they are. To advance high-quality equitable care, the medical field needs approaches to patient engagement that support clinicians' ability to learn from and engage with patients' understandings of health-related phenomena. At the level of patient–provider interaction, cultural humility is a promising example—a practice that acknowledges the dynamic and complex nature of patient “culture,” including the variation in practices, values, and beliefs within social groups.^68,69^

Recognizing that no course or workshop will equip providers to become completely “competent” in any given group's culture, cultural humility emphasizes the uniqueness of each patient and the importance of clinicians' active, ongoing efforts to learn from their patients as individuals.^68–71^ Cultural humility trainings are not yet widely implemented nor evaluated in medicine, but some examples show promise.^72,73^ Curricular components include experiential service-learning,^74,75^ simulation,^72,73,76^ and reflexive practices (e.g., journaling).^77,78^ Structural humility,^79,80^ anti-racism,^79^ and implicit bias training,^81,82^ which some states and health facilities now require,^83–85^ may bolster cultural humility skills-building.

Certain models of care may additionally support clinicians' deeper understanding of patient perspectives. Group-based care, for example, allows for longer discussions about patient perspectives and their role in patients' behaviors and decision making.^86,87^ Similarly, engaging non-clinicians such as doulas and community health workers,^88,89^ who develop longitudinal trusted relationships with patients, may help the larger care team to better understand, appreciate, and engage with patient perspectives.^73,74^ As states expand coverage,^90,91^ these essential workers will play a greater role in bridging the historically disparate social worlds of community members and clinicians. Finally, health care leadership can support clinician cultural humility by ensuring institutional practices, resources, trainings, and mission statements are consistent with its principles.^68,92^

### Limitations

The primary limitation of this study is its generalizability and the age of the data. The sample was a purposive sample in Northern CA in 2009–2011; patterns of exposure to specific ideas may not represent any one public or private institution and may have shifted. New research should be done to further explore and characterize the differences reported here. However, the existence of differences across patient populations in the US's heterogeneous health care systems, and of public patients' greater exposure to ideas about risk and limited autonomy, likely persist: complementary research in other regions and in recent years echo these phenomena.^24,27,28,66^ A second limitation is that the race/ethnicity measure employed in the larger study did not allow for respondents to report their self-identified race and ethnicity in the full range they may have experienced them. Though respondent race/ethnicity is not a focus of this study, it should be interpreted with caution as representing a constrained characterization of respondents' racial/ethnic identities.

## Conclusion

Pregnant and birthing people enter perinatal care having been exposed to a wide range of ideas about self-care, birth care, and infant care, many of which are contradictory and inconsistent with medical advice. Some ideas are more familiar to individuals in public or private settings, including ideas about risk in delivery and diminished autonomy. Greater clinician understanding and appreciation for the social contexts of patients unlike them may support better patient–provider communication and interaction—an outcome desired by patient, advocate, and professional stakeholders. Clinicians should employ approaches to care that allow them to better understand and engage their patients' perspectives to achieve respectful, high-quality, and equitable care.

## Data Availability

Interested parties may contact the first author to inquire about accessing a deidentified dataset.

## Abbreviations Used

BIPOC: Black, indigenous, and other people of color
L&D: labor and delivery
SD: standard deviation

## Appendix

**Appendix Table A1.** Exposure to Contradictory Ideas: Proportion Reporting Having Heard More Than One Idea About a Surveyed Topic, by Payer Group

Topic	Public		Private	
	%	SD	%	SD
A. Physical activity during pregnancy	62.9	0.49	68.5	0.47
B. Factors affecting fetal health^*^	50.0	0.50	37.1	0.49
C. Experience of pregnancy^*^	16.9	0.38	41.9	0.50
D. Drinking during pregnancy^*^	58.1	0.50	83.1	0.38
E. Epidural use^*^	17.7	0.38	50.0	0.50
F. Risk in labor and delivery	24.2	0.43	20.2	0.40
G. The value of breastmilk vs. formula	41.1	0.49	37.9	0.49
H. Nursing boy vs. girl babies^*^	6.5	0.25	0.8	0.09
I. Timing of baby feeding^*^	57.3	0.50	87.1	0.34
J. Breastfeeding as compulsory or optional	31.5	0.47	41.9	0.50
K. Experience of birth^*^	16.1	0.37	40.3	0.49
L. Babies' ability to manipulate^*^	9.7	0.30	30.6	0.46

Note: Categories with an asterisk (^*^) indicate variables differ significantly by payer (*p*<0.05).

SD, standard deviation.
